# A Post Hoc Analysis of Demographic, Socioeconomic, Health and Mental Health Factors Following a Lactation-Consultant-Led Telephone Breastfeeding Support Program

**DOI:** 10.3390/nu18101601

**Published:** 2026-05-18

**Authors:** Wei Qi Fan, Jessica Zhang, Debra Bourne, David Tran

**Affiliations:** 1 Northern Health, Melbourne 3076, Australia; debra.bourne@nh.org.au (D.B.); david.tran2@nh.org.au (D.T.); 2 Faculty of Medicine, Dentistry and Health Sciences, Melbourne University, Melbourne 3010, Australia

**Keywords:** exclusive breastfeeding, breastfeeding cessation, telephone support, maternal risk factors, mental illness

## Abstract

Background/Objectives: Breast Milk Feeding (BMF) benefits mother and infant. However, women with select risk factors report shorter breastfeeding durations. Our previous prospective cohort observational study of a lactation-consultant-led telephone-based support program in the first month postpartum increased BMF rates up to 6 months. This post hoc study further evaluated the program for mothers at increased risk of early breastfeeding cessation. Methods: We performed secondary analysis involving 762 mothers (control, *n* = 378; intervention, *n* = 384), recruited between 2018 and 2019. Infant feeding types, including BMF, were recorded at 1, 3 and 6 months. Feeding outcomes were analyzed in association with maternal risk factors. *p*-values, odds ratios and 95% confidence intervals were reported via both univariate (UVA) and multivariate regression analysis (MVA). Results: Via MVA, the intervention was associated with increased 6-month BMF rates in these groupings [OR (95%CI), *p*-value]: European [1.80 (1.07–2.96), *p* = 0.027]; South Asian [1.93 (1.19–3.13), *p* = 0.008]; employed [1.47 (1.02–2.12), *p* = 0.038]; unemployed [2.15 (1.33–3.50), *p* = 0.002]; married [1.71 (1.22–2.39), *p* = 0.002]; social support present [1.51 (1.05–2.16), *p* = 0.026]; chronic illness [1.93 (1.35–2.75), *p* = 0.001]; gestational diabetes mellitus [2.17 (1.19–3.95), *p* = 0.11]; overweight and obese [1.48 (1.03–2.12), *p* = 0.034]. A derived success score across the study period indicated via UVA associated increases in BMF rates with history of depression and anxiety (MI) [*p* = 0.044] and ongoing MI [*p* = 0.033], but these increases were smaller than that for no history of MI [*p* < 0.001]. No effect was observed in East/Southeast Asian mothers, Middle Eastern mothers, single or de facto mothers, older mothers, mothers without social support and mothers of any skill level. Conclusions: Although early postpartum telephone support was associated with a number of positive findings of improved BMF at 6 months and over the course of the study, the results were mixed. This suggests that future breastfeeding telephone-based initiatives need to be multifaceted in order to target mothers at risk of early breastfeeding cessation.

## 1. Introduction

Breastfeeding has proven reciprocal health benefits for both mother and baby. In Australia, mothers are aware of the benefits of breastfeeding—achieving initiation rates of over 90% [1]. However, duration of exclusive breastfeeding remains suboptimal, with only 37.5% reaching the World Health Organization (WHO) recommendation of at least 6 months [1,2]. Infants exclusively breastfed for the first 6 months postpartum acquire a range of health benefits [3,4], as well as lowered risk of gastrointestinal infection and death. Mothers also have multiple benefits including rapid postpartum weight loss and prolonged lactational amenorrhea [5,6].

The causes of early breastfeeding cessation are multifaceted and multifactorial. While difficult to categorize definitively, they can be broadly viewed as functional (lack of milk supply, nipple attachment, pain), socio-demographic (maternal age, education level, income, marital status), psychological and social (depression, mental illness, social support), physical and medical (obesity, chronic illness, cesarean section) and cultural (mother and extended family expectations, community preference for artificial feeding, family income support) [7,8,9,10].

Given the complexity of the concern, the Healthcare Sector and especially Public Health have developed a range of strategies aimed at improving breastfeeding rates in the first 6 months of infant life. In general, the duration of breastfeeding is improved if breastfeeding initiation is high and the support program is delivered via professionally trained staff and is scheduled and tailored to local conditions [11]. Of the available support modalities, telephone-based interventions fulfill an important niche [12] as they represent a cost-effective alternative for hospitals while being convenient and accessible for patients.

Between 2018 and 2020 we designed and carried out a prospective cohort observational study of a lactation-consultant-led telephone-based support program (LCTSP) in the first 3 weeks postpartum, which observed 1-, 3- and 6-month breastfeeding rates and evaluated these against risks to early breastfeeding cessation in our local community. Our local Australian community is situated in the outer suburban area of a major city, is multi-cultural, multi-ethnic and of lower socioeconomic status [13]. We reported our findings mainly related to functional and physical/medical categories of known early breastfeeding cessation risk factors. In general, there was a very positive response to the lactation consultant support program and breastfeeding rates improved over the 6-month period. However, due to funding restrictions at that time we were not able to evaluate further.

Subsequently, we carried out a post hoc analysis of our previously published prospective cohort observational study in order to further evaluate other at-risk categories of early breastfeeding cessation including socio-demographic, psychological/social and cultural aspects. We hypothesized that, given both the diversity of our community and the complexity of factors influencing early breastfeeding cessation, the response to our support program would be mixed and the evaluation may reveal aspects requiring prioritization and further research.

## 2. Materials and Methods

### 2.1. Overview

The current study represents a secondary data analysis of a prospective cohort observational study originally conducted from February 2018 to October 2019 at a major public teaching hospital serving an outer suburban, ethnically diverse, multi-socioeconomic region of Melbourne, Australia. This study observed whether there was an improvement in mother–infant pairs at risk of early breastfeeding cessation in breastfeeding rates for up to 6 months postpartum following a lactation-consultant-led, telephone-based support program. Included were mothers with healthy infants who had commenced breastfeeding upon postnatal discharge and were English-speaking. The control group (*n* = 379) received current, standard, established counseling and postnatal and post-discharge care. The exposure group (*n* = 386) received proactive outpatient breastfeeding standard care via telephone contact weekly in the first 3 weeks following birth. All calls were made by International Board-Certified Lactation Consultants and followed a structured questionnaire. During these phone calls, the lactation consultant enquired about areas of concern, providing personalized breastfeeding care. Feeding status was also recorded. To assess feeding status, mothers in both groups then received follow-up telephone contact at 1, 3 and 6 months. Telephone contact was discontinued when mothers were no longer expressing. This study was granted ethics approval by the Austin Health Human Research Ethics Committee, which operates in accordance with the Australian National Health and Medical Research Council’s National Statement on Ethical Conduct in Human Research, HREC Reference number: LNR/17/Austin/371. A detailed description of the methods used in the original study is provided elsewhere [13].

The purpose of the current study was to further explore the aim (as detailed above) in the context of demographic and socioeconomic determinants of health. Also investigated was the impact of maternal diagnosed anxiety and depression as the original study had observed improvements in breastfeeding rates amongst mothers with depression as assessed via the Edinburgh Postnatal Depression Scale (EDPS) [14]. Of interest were factors such as non-English-speaking background, maternal employment, single parenthood, social support level, and medical and mental illness.

### 2.2. Data Collection

Data relating to the mother’s biological, psychological and social characteristics was extracted from the Northern Health Clinical Patient Folder, Birthing Outcomes Summary, Patient Manager. Psychological characteristics included history of mental illness, active mental illness and highest recorded EDPS score. Social characteristics included mother’s ethnicity, country of birth, Aboriginal or Torres Strait Islander status, occupation, marital status, social support, documented social concerns (housing instability, financial disadvantage, domestic issues) and region of residence.

### 2.3. Definitions

Chronic illness was defined as the documented history of a long-lasting health condition requiring ongoing medical attention which also limits activities of daily living, which has the potential to exert disease burden postpartum. Conditions reported in medical records included asthma, anemia, type 2 diabetes mellitus, polycystic ovarian syndrome, thyroid disorders, endometriosis, arthritis, autoimmune disorders, cardiac disorders and sleep disorders. Obstetric conditions were excluded from this category due to their typical resolution following delivery.

Mental illness (MI) was defined as a clinically diagnosed psychiatric condition present during pregnancy. For this study the conditions were limited to major depressive disorder (MDD) and anxiety disorders (Ax), or a combination of both This analysis focused on women with established mental health diagnoses, rather than EDPS screening scores as reported in the original study [13].

Social support was defined as the documented presence of at least one close family member or friend—excluding the partner—as a source of maternal support providing psychological, emotional, and material assistance. This definition was selected in attempt to differentiate social support from the impact of marital status.

Antenatal Model of Care (AMC) refers to the different ways maternity care is delivered. At a service level, AMC describes the location and the way care is provided, which carers are involved and the continuity of care. AMC is evaluated by via risk levels (low to high), carer continuity, and mother–baby clinical outcomes. Initial effective risk assessment occurs at booking and continuously throughout the antenatal period [15].

Occupations were stratified into Skill Levels 1–2 according to the Australian and New Zealand Standard Classification of Occupations (ANZSCO), which grossly correlates with the level of formal education required [15]. As maternal education attainment was not routinely documented in medical records, occupational skill level was used as a proxy measure of education to allow exploration of its association with breastfeeding outcomes. ANZSCO Occupation Skill Level 1 represents a level of skill commensurate with a bachelor’s degree or higher qualification. At least five years of relevant experience may substitute for the formal qualification. ANZSCO Occupation Skill Level 2 represents a level of skill commensurate with one of the following: New Zealand Register Diploma or Australian Qualifications Framework Associate Degree, Advanced Diploma or Diploma. At least three years of relevant experience may substitute for the formal qualifications listed above [16].

In this report, the term “Breast Milk Feeding” (BMF) adheres to the WHO definition: “The infant receives only breast milk. No other liquids or solids are given—not even water—with the exception of oral rehydration solution, or drops/syrups of vitamins, minerals or medicines” [17]. BMF refers to either direct breastfeeding or providing expressed breast milk via bottle or feeding tube and was used in the original study [13]. Artificial feeding (AF) was defined as the use of commercial, manufactured milk designed for infant nutrition. Mixed feeding (MF) was defined as a combination of both BMF and AF.

### 2.4. Statistical Analysis

Normality was assessed using the Shapiro–Wilk test. Descriptive statistics were calculated using Mann–Whitney U for non-normal continuous variables and Chi-square test for categorical variables. Univariate Chi-square and Fisher’s Exact tests were used to compare breastfeeding outcomes between control and exposure groups, stratified by variables associated with early breastfeeding cessation. Odds ratios (ORs) and associated 95% confidence intervals (CIs) were reported. In assessing the impact of MI and for co-morbidities, due to relatively low numbers of cases, BMF data was pooled across the 6 months of the study. It should be noted that once a mother had ceased breastfeeding either at 1 or 3 months, no further data was collected. Essentially, an ordinal score (0, 1, 2, or 3) was calculated that represents the total duration of success for each individual, a common approach to summarizing longitudinal binary data when the outcome is irreversible (monotonic). To control for confounders, multivariate regression was performed on clinically important parameters found to be statistically significant by univariate analysis. Adjusted ORs, 95% CIs and *p*-values were reported. To test for false positives due to multiple-comparison testing, Holm’s, Hotchberg’s and Benjamini–Hochberg tests were performed. Statistical significance was set at *p* < 0.05. Data was analyzed using IBM SPSS Statistics for Windows, Version 30 (IBM Corp., Armonk, NY, USA).

## 3. Results

### 3.1. Patient Population

Though the original cohort recruited 765 mothers (control, *n* = 379; exposure, *n* = 386), analysis was performed for a total of 762 participants (control, *n* = 378; exposure, *n* = 384). Three records were excluded due to missing data.

### 3.2. Baseline Characteristics

Table 1 presents participants’ baseline characteristics for the maternal factors evaluated in this study. No statistically significant differences were observed between control and exposure groups. Not reported in this table is breastfeeding initiation rates. Initiation rates were extremely high with only five instances of the first feed being formula across both cohorts.

### 3.3. BMF Rates by Demographic and Socioeconomic Characteristics

Table 2 presents BMF data at 6 months. Data for the 1- and 3-month periods is listed in Appendix B.

European and South Asian mothers had increased BMF rates associated with the LCTSP at 6 months and also at 1 and 3 months. However, this was not the case with East and Southeast Asian mothers, with no improvement at the three time points, nor with Middle Eastern mothers. It should be noted that East and Southeast Asian mothers had lower participation rates in the LCTSP.

Not reported in Table 2 is the general comparison between immigrant and Australian-born women. In both cases the LCTSP was associated with increases in BMF at 6 months (all *p* < 0.05) with the immigrant cohort having marginally higher ORs.

There was no LCTSP-improved response over control for single or de facto mothers. Married mothers had associated improvements in BMF at 6 months and earlier.

Mothers with higher or lower skill levels did not respond to the LCTSP with increased BMF at 6 months.

With regard to either employment status, the LCTSP was associated with a positive influence on BMF at 6 months.

### 3.4. BMF Rates by Determinants of Health Factors

Table 3 presents BMF data at 6 months. Data for the 1- and 3-month periods is listed in Appendix C. There was evidence of a direct relationship between maternal age and improved BMF across the 6-month period in response to the LCTSP. For mothers 24 years or younger, there was an improvement in BMF only at 1 month. For mothers between the age of 25 and 34 years, associated improvement was observed at 1 and 6 months, while mothers 35 years and older had associated improvements in BMF rates at all time periods.

Regardless of BMI category, BMF rates improved over control at 6 months.

**Figure A1:**
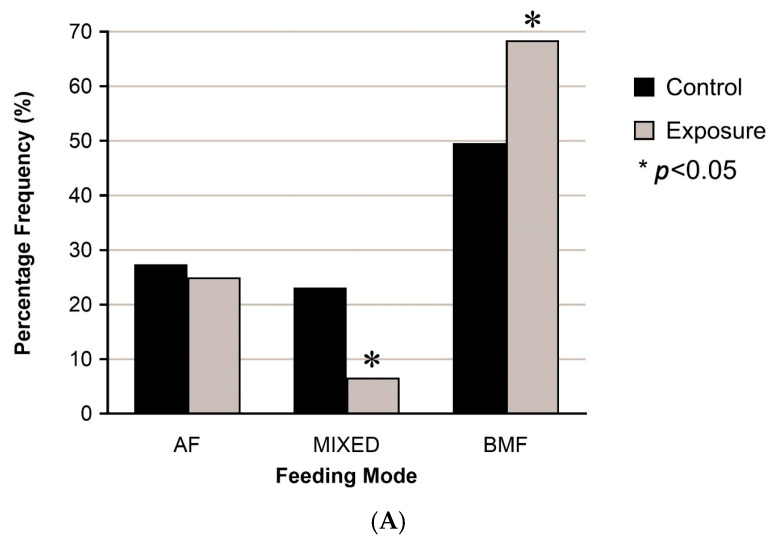

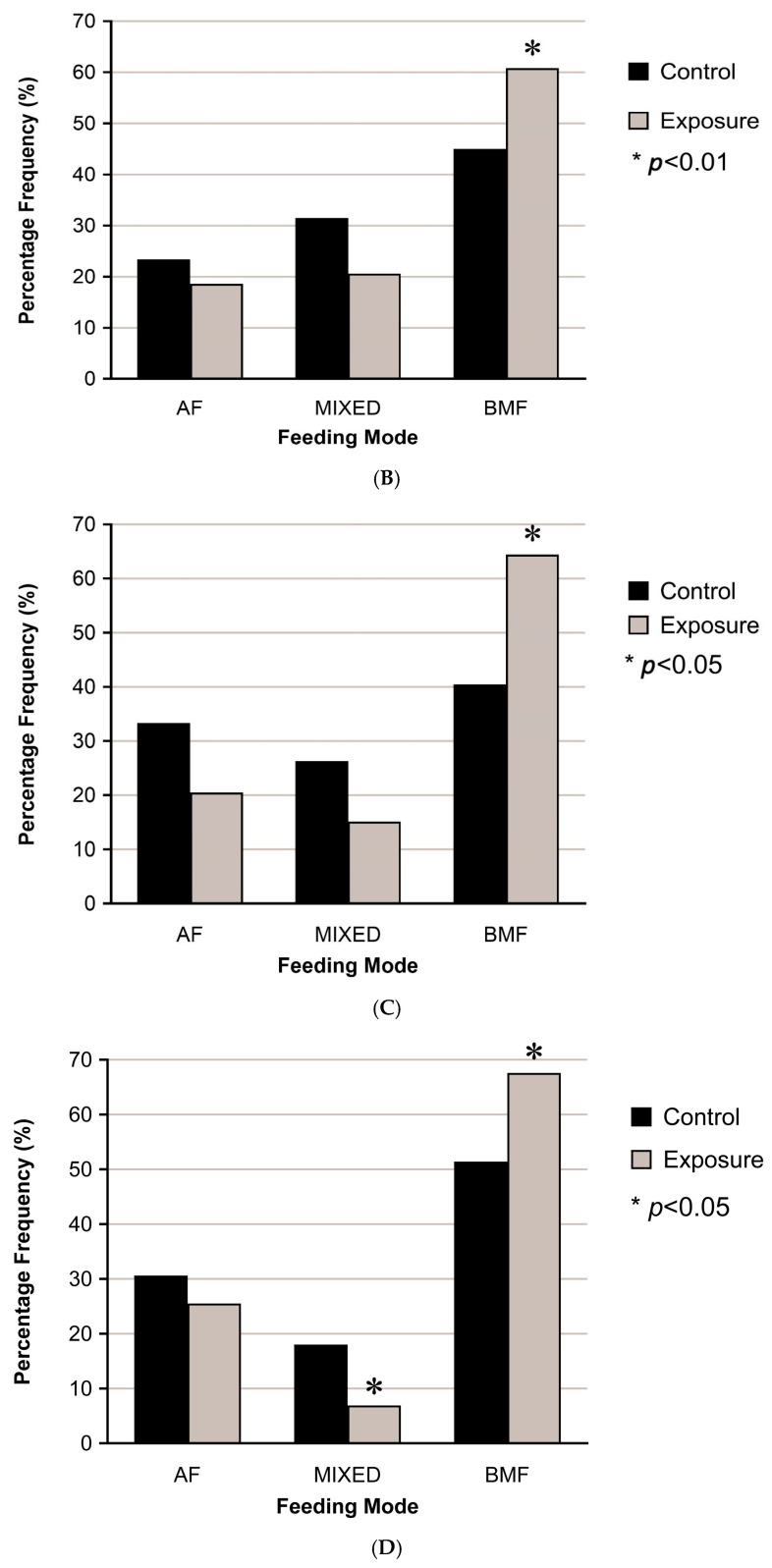
(**A**) Overall feeding outcomes for mothers with anemia. Total number of data points across 6 months (control:exposure): (117:76). BMF rates across 6 months: 49.6% vs. 68.4%, *p* = 0.012. (**B**) Overall feeding outcomes for mothers with hypothyroidism, hyperthyroidism or goiter. Total number of data points across 6 months (control:exposure): (111:102). BMF rates across 6 months: 45.0% vs. 60.8%, *p* = 0.028. (**C**) Overall feeding outcomes for mothers with PCOS. Total number of data points across 6 months (control:exposure): (57:73). BMF rates across 6 months: 40.4% vs. 64.4%, *p* = 0.008. (**D**) Overall feeding outcomes for mothers with asthma. Total number of data points across 6 months (control:exposure): (111:102). BMF rates across 6 months: 51.4% vs. 67.6%, *p* = 0.018.

For mothers with and without chronic disease or gestational diabetes, there was a positive BMF response associated with the LCTSP compared with control at 6 months. Figure A1A–D, Appendix A, presents univariate data for chronic disease categories of anemia, thyroid disorder, asthma, and polycystic syndrome—all indicating an aggregate improvement (see Section 3.5 below) in BMF across the 6 months of the study period.

The response to the LCTSP compared to control was similar for both mothers referred to the Antenatal Care Model with a medium- to high-risk category and mothers having pre-discharge lactation consultant referrals with BMF improvements at 6 months.

Mothers regardless of social support had a positive response to the LCTSP with improved BMF at 6 months.

### 3.5. The Impact of Mental Illness on BMF Rates Following the LCTSP

In the original study [13], the impact of maternal depression was observed. Due to fewer numbers of such cases, an aggregate approach was adopted where all data points across 1, 3 and 6 months were included to improve precision and reliability (refer also to Section 2.4). This approach has been continued in the current supplementary study.

#### 3.5.1. Overall Impact on BMF Rates of MI

**Figure 1:**
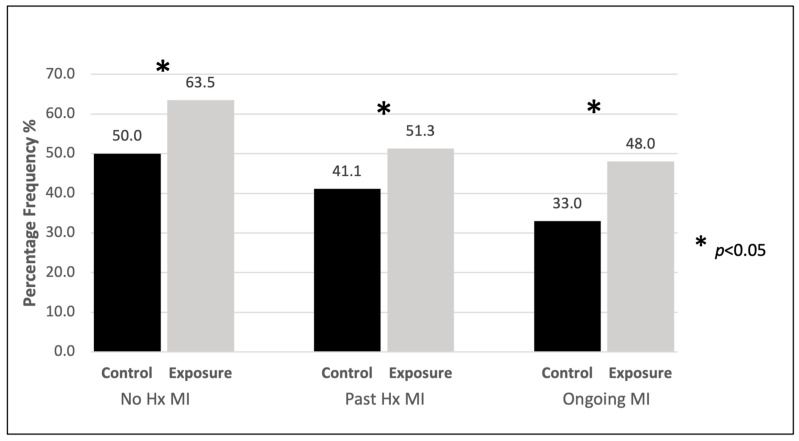
Overall BMF outcomes for mothers with and without MI (MDD and/or Ax). Total number of BMF data points across 6 months (control:exposure): no history of MI (806:769) *p* < 0.001; past history of MI (197:195) *p* = 0.044; ongoing MI/active MI including a past history of MI (106:100) *p* = 0.033. (Hx—history).

Figure 1 illustrates the impact of diagnosed MI (MDD and Ax) on BMF rates following the LCTSP. Statistics are from univariate analysis. While there is an associated increase in the frequency of BMF across the 6-month study period, mothers with MI had comparatively lower frequencies than for mothers with no history of MI.

#### 3.5.2. Multivariate Regression Analysis

Table 4 presents regression analysis outcomes on BMF data at 6 months for clinically important parameters found to be statistically significant by univariate analysis in Table 2 and Table 3. Despite the inclusion of multiple relevant confounders, the majority of univariate findings were supported—with the exception of maternal age above 24 years and the absence of social support.

#### 3.5.3. Testing for False Positives

As this study performed multiple-comparison testing on the database, the possibility of false-positive results was addressed using the comparisons done via multivariate regression analysis as a basis. The conservative Holm–Bonferonni method and Hotchberg method calculated a *p*-value of ≥0.026 as non-significant. However, the Benjamini–Hotchberg method calculated *p* > 0.05 as non-significant.

## 4. Discussion

A review of the literature reveals multiple studies detailing risk factors for breastfeeding cessation and follow-up studies aimed at providing intervention strategies to improve breastfeeding rates, especially up to the WHO recommended length of 6 months postpartum [2]. Many studies use clever and innovative approaches to promote breastfeeding from perceived causal factors. Approaches include trained peer-group support [18], use of technology such as smart phone apps [19], education programs starting in the antenatal period [20] and telephone-based postnatal help [21]. Given that the successful continuation of breastfeeding after birth arguably relies on overcoming a multiplicity of maternal, demographic, socioeconomic, cultural and health-related risks, it is not surprising that breastfeeding interventionist studies have had limited or mixed success. However, many of the published studies, while reporting on basic characteristics and breastfeeding rates, provide limited analysis of the impact of the intervention on relevant risk factors. This current post hoc analysis is a follow-up on our previously published study [13] which reported improved breastfeeding for up to 6 months following a postnatal LCTSP. The original study mainly observed the impact of obstetric and perinatal factors on breastfeeding outcomes. This study has extended the observations to socio-demographic, cultural and health aspects. The authors contend that an understanding of these aspects is pertinent to both an improved appreciation of causal factors (both cessation and continuation of breastfeeding) and to the design and implementation of future programs of breastfeeding promotion.

Our study reports the associated impacts of the LCTSP on maternal demographic and socioeconomic characteristics at 6 months. As mentioned above, the catchment area for our hospital is a lower socioeconomic, multi-cultural community. More than one-third of residents in Australia are born overseas [22]. In this community-based study, over 50% of participants were overseas-born and almost 70% were of non-European background. There was a similar associated improvement in breastfeeding rates for both Australian-born and overseas-born mothers—a finding not observed in another lactation consultant breastfeeding support study [23]. Our study observed mixed success in BMF when participants were stratified by ethnicity. East/Southeast Asian and Middle Eastern mothers experienced no improvement at any time point, while European and South Asian participants had higher breastfeeding rates in response to the LCTSP—via both univariate and multivariate regression analysis.

These findings for East/Southeast Asian and Middle Eastern mothers are not unexpected—data from a 2010–2011 Australian National Infant Feeding Survey showed that South Asian mothers maintained breastfeeding for a median of 5 months versus 3 months for Australian/European mothers [24]. A study of Chinese Australian women showed that although breastfeeding initiation was almost universal, by 1 month postpartum, over half had introduced infant formula—twice the rate of the general population [25]. This preference for early formula introduction is documented among East Asian immigrants [26]. Middle Eastern mothers also demonstrated higher breastfeeding initiation, but low BMF rates [27]. As the original study was limited to English-speaking participants, this may partially explain the discordant BMF rates between the Middle Eastern and East/Southeast Asian cohorts on the one hand and the South Asian and European cohorts on the other. Migrant Arabic and Chinese mothers in Australia navigate infant feeding by relying on traditional networks and trusted bi-cultural GPs, often finding mainstream services poorly understood or culturally insensitive and having to manage conflicting advice from peers, online sources, and health professionals [28]. As English fluency is common in South Asia, South Asian and European mothers may have been better positioned to benefit from the English-only LCTSP. While there are cultural pressures amongst South Asians to breastfeed, evidence from the UK suggests that cultural influences on breastfeeding amongst South Asian migrants can be both positive and negative [29] and that there is a negative impact due to acculturation [30].

It is interesting to note that at 6 months, there was no associated improvement in mothers of either skill level due to the LCTSP. This is counter to other findings such as an Australian longitudinal study which reported that women with lower education had reduced breastfeeding duration compared to those of higher education [31] and a US prenatal breastfeeding support initiative which reported increased breastfeeding in higher educated mothers [32]. However, in this study univariate analysis suggested that there was a different pattern in breastfeeding reduction across the 6 months. Breastfeeding frequency amongst lower skilled mothers reduced by around 15% across the 6 months, compared to a 30% reduction in the higher skilled mothers. Caution should be assumed in interpreting these findings as demographic data on actual education levels was not recorded in the medical records, making skill level determination difficult.

An important contribution to the literature is our findings relating to maternal determinants of health factors in response to the LCTSP, as there appears to be little relevant published information, especially in regard to maternal co-morbidities. Univariate analysis suggested a proportional relationship with improved breastfeeding response following the LCTSP from younger to older mothers. This finding is consistent with a breastfeeding survey in another culturally diverse and lower socioeconomic community in Australia which reported prolonged breastfeeding in older maternal age [33]. However, multivariate regression analysis did not support the contention that intervention improved breastfeeding at 6 months in older women. An interesting finding of our study was that at 6 months, mothers, regardless of BMI status, had associated improvements in breastfeeding rates over the control cohort via both univariate and multivariate analysis. Due to obesity being a known risk factor for breastfeeding with reported rates lower than for non-obese mothers [34], we previously reported improved obesity-related breastfeeding rates for this study [13]. That the LCTSP was associated with improved breastfeeding rates is pleasing due to the considerable maternal and infant benefits of breastfeeding [35]. The associated response to the LCTSP was positive for mothers with chronic disease generally and in particular for those with gestational diabetes (Table 2 and Table 4). Univariate analysis also suggested a positive response for anemia, thyroid concerns, polycystic ovary syndrome and asthma (Figure A1A–D, Appendix A), but low data numbers prevented further analysis. An improvement in breastfeeding rates at 6 months postpartum has also been reported for a breastfeeding educational program for asthma [36]. Previous research has demonstrated associations between maternal social support levels and both BMF rates and breastfeeding self-efficacy [37,38]. Social support is clearly important to breastfeeding success as mothers prioritize breastfeeding advice from relatives, followed by friends, over formal breastfeeding support [39]. However, across the literature there are varying definitions of social support including: single mothers with no partner [40]; presence of intimacy and social integration [41,42]; inferred social circumstance such as low socioeconomic status or overseas birth [23,43]. In the current study, social support was defined as the presence of at least one close family member or friend—excluding the partner in order to maintain a distinction from partner support. In response to our LCTSP, mothers with existing family or friend support networks demonstrated associated increases via univariate and multivariate analysis in BMF rates at 6 months. However, the intervention was not effective amongst mothers without social support, as assessed by multivariate analysis. Potentially supporting this finding amongst mothers lacking social support, it should be noted that single and de facto mothers also did not have improved breastfeeding rates in our study. However, there are arguably other impacting factors apart from social support such as financial stability, workplace flexibility and time allocation.

Maternal mental illness and BMF exhibit a complex bi-directional relationship where each influences the other. On one hand, pre-existing mental illness physiologically predisposes the mother towards reduced milk production [8] and lowers breastfeeding self-efficacy [44]. Conversely, successful breastfeeding can improve maternal mental health outcomes and there has been some success in breastfeeding programs which include combined mental health counseling with conventional lactation support [45]. In our initial study, based on EPDS scores only, the LCTSP was associated with improved breastfeeding for mothers with depression [13] across the 6-month study period. This study, using diagnosed MI data, suggested that while there was improved BMF frequency following the LCTSP, mothers with MDD and Ax were arguably at a disadvantage compared to mothers with no history of MI who had higher BMF rates. Caution is advised in interpreting these results due to the univariate analysis.

The limitations of this study have been detailed in the original study [13] including the inherent drawbacks of a telephone-based program. Worth mentioning here is that the study was restricted to English-speaking participants and this excluded relevant input from many mothers, especially when evaluating the impact of the LCTSP on ethnic-group breastfeeding. The use of the ANZSCO skill classification system as a proxy for education level in this study is a potential a shortcoming in that current employment does not always reflect true educational attainment. This is particularly relevant in immigrant populations, where individuals who attain higher education often work in roles below their level of training in the new country. This study performed multiple-comparison testing on the database, resulting in the possibility of false positives. To address this the conservative Holm–Bonferonni method and Hotchberg method were performed to assess family-wise error rate (FWER) and the Benjamini–Hotchberg method to assess false discovery rate (FDR). The FWER testing suggested *p*-values ≥ 0.026 to be non-significant, but the FDR testing suggested that the standard significance value of *p* < 0.05 was still valid. FWER testing can lead to missing real effects and FDR testing allows for an expected proportion of false positives among rejected null hypotheses [46]. Consequently, the significance of results in this study with *p*-values between 0.026 and 0.05 should be viewed with this caveat.

Despite the limitations of this study, the authors believe that there are a number of important strengths to be recognized. Firstly, the study cohort size of over 750 participants facilitates adequate numbers for many subgroup analyses. Secondly, although the positive associations are important to evaluate the success of the program, this post hoc analysis helps to identify which specific demographic subgroups and health-related concerns benefitted least from the program. This facilitates the creation of tailored interventions for future, more effective support, particularly to our demographic of low-income women who often face structural barriers to breastfeeding. For our community this would include single mothers, mothers in de facto relationships, those without social support and two large ethnic subgroups of Middle Eastern and East and Southeast Asian mothers. These mothers had little associated impact from the LCTSP of improved breast feeding at 6 months compared to the community control group. All had a language barrier that needs to be addressed in any future LCTSP. Finally, the analysis revealed which areas of concern were more difficult to assess because of low data availability. For this study, the suggestion from univariate analysis that mothers with MI may be at a disadvantage in breastfeeding success compared to mothers without MI is an alert that requires further investigation and targeted strategies—especially as depression and anxiety are common forms of MI encountered in pregnancy and childbirth [47].

## 5. Conclusions

This secondary analysis of data from our original cohort observational study of a lactation-consultant-led telephone-based support program in the first month postpartum provided a number of associations of improved BMF at 6 months and over the course of the study. However, the results were mixed. Importantly, it is the non-significant findings of this study that may inform future directions for telephone-based lactation consultant programs. While the program developed for this study was associated with some significant improvements in breastfeeding, there is a need for further nuanced research and development to improve breastfeeding uptake amongst mothers with demographic and healthcare-related factors associated with earlier breastfeeding cessation.

## Figures and Tables

**Table 1:** Participants’ baseline characteristics.

Characteristics	*Descriptor*	Control (*n* = 378)	Exposure (*n* = 384)	*p*-Values
Maternal age (mean yrs ± SD)		30.4 ± 4.7	29.9 ± 4.9	0.108
Ethnicity	European	91 (30.2%)	104 (34.1%)	
	South Asian	111 (36.9%)	121 (39.7%)	
	East and Southeast Asian	21 (7.0%)	22 (7.2%)	0.312
	Middle Eastern	65 (21.6%)	51 (16.7%)	
	* Other	13 (4.3%)	7 (2.3%)	
Country of Birth	Australia	175 (46.3%)	179 (46.6%)	0.942
Employment Status	Employed	215 (62.3%)	231 (66.6%)	
	Unemployed	122 (35.4%)	105 (30.3%)	0.323
	Student	8 (2.3%)	11 (3.2%)	
Marital Status	Married	287 (75.9%)	271 (70.6%)	
	De Facto	48 (12.7%)	60 (15.6%)	0.252
	Single	43 (11.4%)	53 (13.8%)	
Social Support	Present	238 (86.9%)	272 (91.0%)	0.141
	Absent	36 (13.1%)	27 (9%)	
Chronic Medical Condition	Present	154 (40.7%)	137 (35.7%)	0.157
	Healthy	224 (59.3%)	247 (64.3%)	
	Obesity	105 (27.8%)	91 (23.7%)	0.214
Mental Health	EDPS (mean ± SD) (*n* = 359/355)	6.2 ± 4.6	6.0 ± 4.3	0.501
	History of mental illness	72 (19.0%)	75 (19.5%)	0.927
	Current mental illness	58 (15.3%)	56 (14.6%)	0.839

Data presented as *n* (%) for categorical variables and mean (IQR) for continuous variables. * Includes Sub-Saharan African (*n* = 8), Indigenous Australian (*n* = 7), Polynesian (*n* = 5) and Hispanic (*n* = 2) ethnic groups.

**Table 2:** BMF rates by demographic and socioeconomic characteristics at 6 months.

	Control Group	Exposure Group	OR (95% CI)	*p*-Values
BMF (*n*, %)				
European Ethnicity	30 (37.0%)	49 (54.4%)	2.03 (1.10–3.75)	0.031
South Asian Ethnicity	32 (32.0%)	61 (54.5%)	2.54 (1.45–4.46)	0.001
E or SE Asian Ethnicity	11 (61.1%)	9 (47.4%)	0.57 (0.16–2.12)	0.515
Middle Eastern Ethnicity	20 (32.8%)	20 (46.5%)	1.78 (0.80–3.98)	0.219
Single	15 (38.5%)	19 (40.4%)	1.09 (0.46–2.59)	1.000
De Facto	17 (37.8%)	26 (47.3%)	1.48 (0.66–3.29)	0.418
Married	93 (35.6%)	129 (55.1%)	2.22 (1.55–3.19)	<0.001
Skill Level 1	24 (42.9%)	37 (60.7%)	2.06 (0.98–4.30)	0.065
Skill Level 2	43 (35.5%)	56 (44.8%)	1.47 (0.88–2.46)	0.154
Employed	73 (37.4%)	105 (50.7%)	1.72 (1.16–2.56)	0.009
Unemployed	36 (32.1%)	54 (61.4%)	3.35 (1.87–6.01)	<0.001

**Table 3:** BMF rates by determinants of health factors at 6 months.

	Control Group	Exposure Group	OR (95% CI)	*p*-Values
BMF (*n*, %)				
Maternal age ≤ 24 years	14 (37.8%)	19 (38.0%)	1.01 (0.42–2.42)	1.000
Maternal age 25–34 years	91 (36.7%)	117 (52.5%)	1.90 (1.32–2.75)	<0.001
Maternal age ≥ 35 years	20 (33.3%)	38 (60.3%)	3.04 (1.46–6.35)	0.004
Normal BMI (18.5–24.9)	53 (39.8%)	79 (54.9%)	1.84 (1.14–2.96)	0.016
Overweight BMI (25 ≤ 30)	43 (39.1%)	56 (53.8%)	1.82 (1.06–3.13)	0.039
Obesity BMI (≥30)	27 (28.4%)	36 (43.4%)	1.93 (1.04–3.59)	0.042
Chronic disease—yes	50 (35.5%)	68 (57.6%)	2.48 (1.50–4.09)	<0.001
Chronic disease—no	75 (36.8%)	106 (48.6%)	1.63 (1.10–2.40)	0.018
Gestational diabetes—yes	19 (28.4%)	36 (56.3%)	3.25 (1.57–6.71)	0.001
Gestational diabetes—no	106 (38.1%)	138 (50.7%)	1.67 (1.19–2.35)	0.003
ACM high risk	13 (33.3%)	25 (55.6%)	2.50 (1.03–6.08)	0.050
ACM medium risk	46 (31.3%)	63 (54.8%)	2.66 (1.60–4.41)	<0.001
ACM low risk	66 (41.5%)	86 (48.9%)	1.35 (0.87–2.08)	0.189
LC referral pre-discharge—yes	42 (33.6%)	68 (51.1%)	2.06 (1.25–3.42)	0.006
LC referral pre-discharge—no	83 (37.7%)	106 (52.2%)	1.80 (1.22–2.66)	0.003
Social support present	79 (36.9%)	112 (48.7%)	1.62 (1.11–2.37)	0.013
Social support absent	8 (23.5%)	16 (61.5%)	5.20 (1.70–15.92)	0.004

ACM—Antenatal Care Model. LC—lactation consultant.

**Table 4:** BMF rates at 6 months by multivariate regression analysis of demographic, socioeconomic and health determinant factors. Exposure group compared to control.

	Odds Ratio	95% CI	*p*-Values
European ethnicity	1.80	1.07–2.96	0.027
South Asian ethnicity	1.93	1.19–3.13	0.008
Married	1.71	1.22–2.39	0.002
Employed	1.47	1.02–2.12	0.038
Unemployed	2.15	1.33–3.50	0.002
Over 24 years of age	0.76	0.47–1.23	0.259
Overweight and obese	1.48	1.03–2.12	0.034
Chronic disease	1.93	1.35–2.75	0.001
GDM	2.17	1.19–3.95	0.011
Social support—present	1.51	1.05–2.16	0.026
Social support—absent	2.11	0.80–5.55	0.132

Variables in the regression included: European and South Asian ethnicity—married; chronic disease; MI; parity; social support; Skill Level 2. Married—parity; Australian-born; Skill Level 2; employment; chronic disease; MI; BMI (overweight and obese). Employed and Unemployed: parity; Australian-born; Skill Level 2; social support; chronic disease; MI; overweight and obese. Over 24 years of age: married; employed; parity; chronic disease; MI; social support; overweight and obese. Overweight and obese: married; employed; parity; chronic disease; MI; social support. Chronic disease: married; employed; parity; MI; social support; Australian-born. GDM: married; employed; parity; MI; social support; Australian-born; overweight and obese. Social support present and absent: married; employed; parity; chronic disease; MI; social support; Australian-born; overweight and obese.

## Data Availability

The data that support the findings of this study are not publicly available due to containing information that could compromise the privacy of research participants but are available from the corresponding author upon reasonable request.

## Abbreviations

BMF	Breast Milk Feeding
WHO	World Health Organization
LCTSP	Lactation-consultant-led telephone-based support program
EDPS	Edinburgh Postnatal Depression Scale
AMC	Antenatal Model of Care
ANZSCO	Australian and New Zealand Standard Classification of Occupations
AF	Artificial feeding
MF	Mixed feeding
OR	Odds ratio
CI	Confidence interval
IQR	Interquartile Range
LC	Lactation consultant
MI	Mental illness
Hx	History

## Appendix A

Impact of LCTSP on selected maternal co-morbidities. Figures generated from aggregate data across all time periods—1, 3 and 6 months.

## Appendix B

**Table A1:** BMF rates by demographic and socioeconomic characteristics at 1 and 3 months.

	Control Group	Exposure Group	OR (95% CI)	*p*-Values
BMF (*n*, %)				
European Ethnicity				
1 month	46 (52.3%)	65 (73.9%)	2.58 (1.37–4.86)	0.005
3 months	36 (45.6%)	56 (62.2%)	1.96 (1.06–3.64)	0.032
South Asian Ethnicity				
1 month	56 (51.4%)	84 (72.4%)	2.48 (1.43–4.32)	0.001
3 months	48 (45.7%)	68 (61.3%)	1.88 (1.09–3.23)	0.029
E or SE Asian Ethnicity				
1 month	13 (61.9%)	15 (71.4%)	1.54 (0.42–5.61)	0.744
3 months	14 (77.8%)	11 (57.9%)	0.39 (0.09–1.65)	0.295
Middle Eastern Ethnicity				
1 month	36 (60.0%)	34 (79.1%)	2.52 (1.03–6.18)	0.054
3 months	36 (55.4%)	22 (48.9%)	0.77 (0.36–1.65)	0.562
Single				
1 month	23 (57.5%)	34 (75.6%)	2.29 (0.91–5.76)	0.106
3 months	21 (51.2%)	18 (42.9%)	0.71 (0.30–1.70)	0.512
De Facto				
1 month	23 (48.9%)	36 (66.7%)	2.09 (0.93–4.67)	0.105
3 months	16 (36.4%)	27 (50.0%)	1.75 (0.78–3.95)	0.221
Married				
1 month	155 (56.2%)	179 (73.4%)	2.15 (1.49–3.11)	<0.001
3 months	137 (51.5%)	148 (61.2%)	1.48 (1.04–2.11)	0.032
Skill Level 1				
1 month	38 (65.5%)	47 (74.6%)	1.55 (0.71–3.39)	0.322
3 months	35 (63.6%)	37 (62.7%)	0.96 (0.45–2.06)	1.000
Skill Level 2				
1 month	67 (50.8%)	96 (76.2%)	3.10 (1.82–5.29)	<0.001
3 months	53 (42.4%)	75 (59.1%)	1.96 (1.19–3.23)	0.012
Employed				
1 month	115 (55.0%)	158 (74.5%)	2.39 (1.58–3.61)	<0.001
3 months	97 (48.7%)	124 (59.9%)	1.57 (1.06–2.33)	0.028
Unemployed				
1 month	65 (55.6%)	65 (71.4%)	2.00 (1.12–3.58)	0.021
3 months	55 (47.8%)	52 (55.9%)	1.38 (0.80–2.40)	0.267

## Appendix C

**Table A2:** BMF rates by determinants of health factors at 1 and 3 months.

	Control Group	Exposure Group	OR (95% CI)	*p*-Values
BMF (*n*, %)				
Maternal age ≤ 24 years				
1 month	18 (48.6%)	37 (72.5%)	2.79 (1.15–6.80)	0.027
3 months	16 (43.2%)	20 (40.0%)	0.88 (0.37–2.07)	0.827
Maternal age 25–34 years				
1 month	154 (59.2%)	165 (72.4%)	1.80 (1.23–2.64)	0.003
3 months	134 (52.8%)	136 (59.9%)	1.34 (0.93–1.92)	0.119
Maternal age ≥ 35 years				
1 month	29 (43.9%)	47 (73.4%)	3.53 (1.69–7.38)	<0.001
3 months	24 (40.0%)	37 (60.7%)	2.31 (1.12–4.79)	0.029
Normal BMI (18.5–24.9)				
1 month	83 (58.5%)	108 (74.5%)	2.08 (1.26–3.42)	0.004
3 months	74 (55.2%)	91 (63.6%)	1.42 (0.88–2.30)	0.178
Overweight BMI (25 ≤ 30)				
1 month	70 (61.4%)	86 (79.6%)	2.46 (1.35–4.48)	0.003
3 months	58 (51.8%)	61 (58.7%)	1.32 (0.77–2.26)	0.340
Obesity BMI (≥30)				
1 month	44 (44.0%)	50 (61.7%)	2.05 (1.13–3.73)	0.025
3 months	38 (38.4%)	38 (45.2%)	1.33 (0.74–2.39)	0.370
Chronic disease—yes				
1 month	75 (51.0%)	91 (75.8%)	3.01 (1.78–5.11)	<0.001
3 months	70 (47.6%)	72 (60.0%)	1.65 (1.01–2.69)	0.049
Chronic disease—no				
1 month	126 (58.3%)	158 (70.9%)	1.74 (1.17–2.58)	0.007
3 months	104 (51.0%)	121 (55.5%)	1.20 (0.82–1.76)	0.380
Gestational diabetes—yes				
1 month	32 (43.8%)	47 (70.1%)	3.01 (1.50–6.05)	0.002
3 months	29 (40.8%)	37 (57.8%)	1.99 (1.00–3.94)	0.059
Gestational diabetes—no				
1 month	169 (58.3%)	202 (73.2%)	1.95 (1.37–2.79)	<0.001
3 months	145 (51.8%)	156 (56.9%)	1.23 (0.88–1.72)	0.233
ACM high risk				
1 month	22 (50.0%)	34 (77.3%)	3.40 (1.36–8.53)	0.014
3 months	20 (48.8%)	24 (55.8%)	1.33 (0.56–3.13)	0.662
ACM medium risk				
1 month	82 (52.6%)	88 (71.0%)	2.21 (1.34–3.63)	0.002
3 months	68 (45.0%)	64 (55.2%)	1.50 (0.92–2.44)	0.110
ACM low risk				
1 month	97 (59.5%)	127 (72.6%)	1.80 (1.14–2.84)	0.012
3 months	86 (54.1%)	105 (58.7%)	1.20 (0.78–1.85)	0.442
LC referral pre-discharge—yes				
1 month	62 (47.0%)	100 (73.0%)	3.05 (1.83–5.08)	<0.001
3 months	61 (48.8%)	77 (55.8%)	1.32 (0.82–2.15)	0.268
LC referral pre-discharge—no				
1 month	139 (60.2%)	149 (72.3%)	1.73 (1.16–2.59)	0.009
3 months	113 (50.0%)	116 (58.0%)	1.38 (0.94–2.03)	0.119
Social support present				
1 month	119 (52.4%)	170 (71.4%)	2.27 (1.55–3.33)	<0.001
3 months	108 (49.3%)	126 (54.3%)	1.22 (0.84–1.77)	0.301
Social support absent				
1 month	17 (48.6%)	23 (79.3%)	4.06 (1.33–12.40)	0.019
3 months	10 (30.3%)	16 (16.5%)	3.68 (1.25–10.88)	0.020

ACM—Antenatal Care Model. LC—lactation consultant.
